# *Semi-Automatic In Silico* Gap Closure Enabled *De Novo* Assembly of Two *Dehalobacter* Genomes from Metagenomic Data

**DOI:** 10.1371/journal.pone.0052038

**Published:** 2012-12-21

**Authors:** Shuiquan Tang, Yunchen Gong, Elizabeth A. Edwards

**Affiliations:** 1 Department of Chemical Engineering and Applied Chemistry, University of Toronto, Toronto, Ontario, Canada; 2 Centre for the Analysis of Genome Evolution and Function, University of Toronto, Toronto, Ontario, Canada; University of Georgia, United States of America

## Abstract

Typically, the assembly and closure of a complete bacterial genome requires substantial additional effort spent in a wet lab for gap resolution and genome polishing. Assembly is further confounded by subspecies polymorphism when starting from metagenome sequence data. In this paper, we describe *an in silico* gap-resolution strategy that can substantially improve assembly. This strategy resolves assembly gaps in scaffolds using pre-assembled contigs, followed by verification with read mapping. It is capable of resolving assembly gaps caused by repetitive elements and subspecies polymorphisms. Using this strategy, we realized the *de novo* assembly of the first two *Dehalobacter* genomes from the metagenomes of two anaerobic mixed microbial cultures capable of reductive dechlorination of chlorinated ethanes and chloroform. Only four additional PCR reactions were required even though the initial assembly with Newbler v. 2.5 produced 101 contigs within 9 scaffolds belonging to two *Dehalobacter* strains. By applying this strategy to the re-assembly of a recently published genome of *Bacteroides*, we demonstrate its potential utility for other sequencing projects, both metagenomic and genomic.

## Introduction

The value of assembling complete closed bacterial genomes has been questioned [1] considering the high cost of genome finishing (closing assembly gaps and genome polishing). In the age of Sanger sequencing, when most assembly gaps were caused by insufficient sequencing, the only way to resolve gaps was to perform additional targeted sequencing from the contig ends [2], [3], which is a labor-intensive and costly process. Even with next-generation sequencing (NGS) techniques (454 pyrosequencing, Illumina, SOLiD and others) that provide ample sequence coverage for single microbial genomes, the volume of data and small read length make finishing difficult and time-consuming [4], and thus many genomes remain as drafts.

In the assembly of sequencing data derived from single organisms, the major cause of assembly gaps is the presence of repetitive elements, such as the genes of transposases and reverse transcriptases [5]. A powerful way to resolve such gaps is by incorporating mate-pair sequencing data, and several genome assemblers have incorporated mate-pair constraints into assembly. New stand-alone gap resolution programs, including IMAGE [6], GapResolution (http://www.jgi.doe.gov/software/) and GapFiller [7] were designed to close gaps using mate-pair data. However, gap resolution in the assembly of NGS data is still challenging. In the assembly of the recently published genome of *Bacteroides salanitronis*, sequenced by 454 and Illumina sequencing, 193 additional PCR reactions and 4 shatter libraries were required to close the gaps after the application of GapResolution [8].

In the assembly of metagenomic data, the challenge is compounded with subspecies polymorphism (or strain variation), resulting in fragmentation even in the assembly of non-repetitive genes. The interferences caused by sequences of coexisting similar genomes can cause severe fragmentation in metagenomic assembly. Before 2011, all genome assemblers and gap-resolution programs were designed to handle sequencing data derived from single genomes; therefore they are powerless in resolving the gaps caused by subspecies polymorphism. Newer assemblers, Meta-IBDA [9], Genovo [10], Bambus 2 [11], have been developed recently to address specific challenges faced in metagenomic assembly. With respect to subspecies polymorphism, Meta-IBDA [9] proposes to improve the assembly by condensing the interfering sequences from subspecies organisms into consensus sequences. However, sequence condensation results in the loss of information and the danger of inadvertent frame shifts.

In this paper, we proposed a strategy for *in silico* gap resolution that is capable of resolving assembly gaps within scaffolds caused by repetitive elements and subspecies polymorphisms. This strategy closes assembly gaps using pre-assembled contigs followed by verification with careful read mapping. Applying this strategy to the assembly of two coexisting *Dehalobacter* genomes in a metagenomic context, we were able to resolve nearly all assembly gaps *in silico* and close the genomes. By then incorporating sequencing data from a second metagenome that has only one of the two *Dehalobacter* genomes, the two genomes were successfully separated and polished into finished genomes. The *Materials and Methods* section describes the overall approach taken to obtain and assemble genomes, while the *Results* section provides specific step-by-step details of the procedure and assembly. The *Discussion* and *Conclusion* provide a summary and comparison to other approaches, and recommendations for those considering metagenome sequencing.

## Materials and Methods

### Culture Description and Metagenomic DNA Sequencing

**Figure 1:**
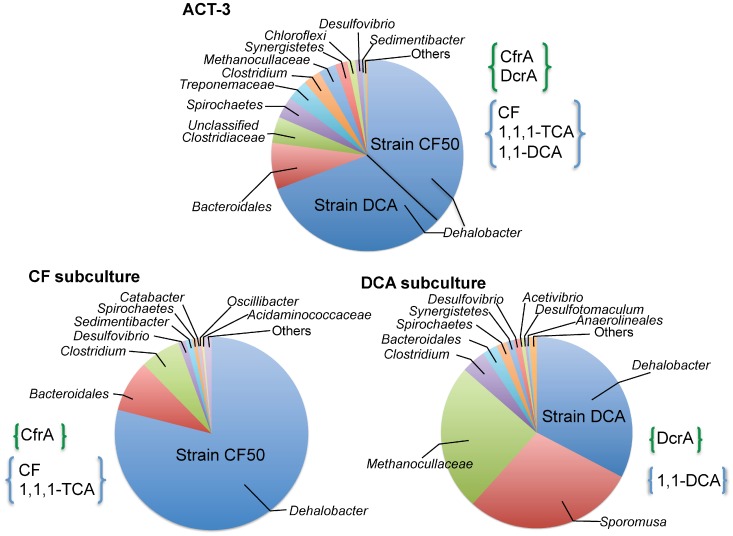
Microbial composition determined by 16S rRNA pyrotag sequences from the ACT-3 parent culture and two subcultures. The ACT-3 culture contains two *Dehalobacter* strains, strain CF50 and strain DCA. Strain CF50 was inherited by the CF subculture, expressing reductive dehalogenase CfrA, which dechlorinates CF and 1,1,1-TCA. Strain DCA was inherited by the DCA subculture, expressing reductive dehalogenase DcrA, which dechlorinates 1,1-DCA. The microbial composition was determined by pyrotag sequencing of the 16S rRNA gene [14].

ACT-3 is an anaerobic enrichment culture that reductively dechlorinates chlorinated ethanes and methanes, including the industrial solvents and groundwater pollutants 1,1,1-trichloroethane (1,1,1-TCA), 1,1-dichloroethane (1,1-DCA), and trichloromethane (or chloroform, CF) [12], [13]. The name of the culture is derived from the contaminants it degrades: “ACT” is TCA backwards, and “3” is the number of chlorine substituents on a single carbon atom in both 1,1,1-TCA and CF; hence the name ACT-3. ACT-3 has been maintained in the laboratory for over a decade in a defined medium with 1,1,1-trichloroethane (1,1,1-TCA) as electron acceptor and a mixture of methanol, ethanol, acetate and lactate as electron donors [12]. Two subcultures enriched with different chlorinated substrates were derived from ACT-3: the CF subculture was grown on chloroform and a mixture of methanol, ethanol and lactate and the DCA subculture was grown on 1,1-DCA and a mixture of methanol, ethenal, acetate and lactate. While the parent culture dechorinates 1,1,1-TCA, 1,1-DCA and CF, the CF subculture only dechlorinates 1,1,1-TCA and CF and the DCA subculture only dechlorinates 1,1-DCA. The community of these three cultures was found to be diverse yet dominated by *Dehalobacter* (Figure 1). The identification of two different but highly similar reductive dehalogenases from these three cultures [14] demonstrated the existence of two *Dehalobacter* strains in ACT-3, one of them was inherited by the CF subculture and the other one was inherited by the DCA subculture (Figure 1), which agrees perfectly with our genome assemblies described herein.

DNA from the ACT-3 parent culture and the CF subculture were sequenced. DNA from the ACT-3 culture was extracted by a CTAB protocol as required by Joint Genome Intitute (JGI) (http://my.jgi.doe.gov/general/protocols/DNA_Isolation_Bacterial_ CTAB_Protocol.doc). This DNA sample was sequenced by the JGI in two runs of 454 pyrosequencing including one mate-pair library with an insert size of ∼8.6 kb. The collection of all the 454 pyrosequencing data from the ACT-3 culture is referred to as the “ACT-3 metagenome”. DNA from the CF subculture was extracted using the UltraClean™ soil DNA isolation kit (MOBIO). The DNA sample was sequenced by the Toronto Center of Applied Genomics (TCAG, Toronto, CA) using mate-pair Illumina sequencing with an insert size of ∼647 bp and read length of 76 bp. The collection of Illumina sequencing data from the CF subculture is referred to as the “CF metagenome”. All raw sequence data have been deposited in NCBI Sequence Read Archive (SRA): SRR554404, 454 paired-end reads of the ACT-3 metagenome; SRR554406, 454 non-paired-end reads of the ACT-3 metagenome; SRR554411, Illumina paired-end reads of the CF metagenome.

### Genome Assembly and Gap Resolution

**Figure 2:**
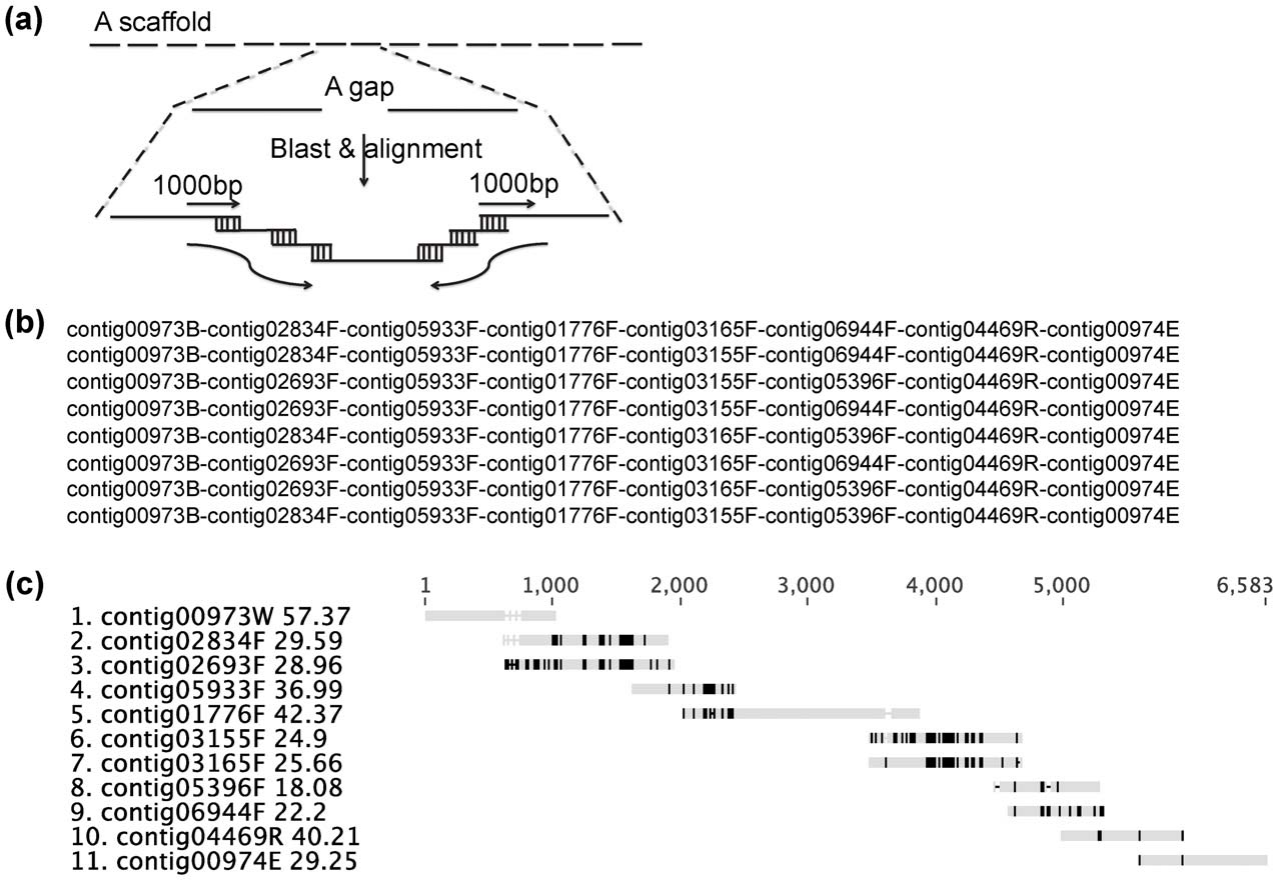
Overview of the *in silico* gap-resolution process. (a) The principle of the perl program that automates the search for overlapping contigs that close an assembly gap. (b) A typical output of the perl program; shown is the case of gap 00973-G-00974; (c) The solutions to gap 00973-G-00974 represented as a multiple sequence alignment created and visualized with Geneious Pro.

The 454 sequencing data of the ACT-3 metagenome were pooled together and assembled by the JGI using Newbler v. 2.5 [15]. A collection of contigs and scaffolds resulted, which can be accessed with IMG taxon object ID of 2100351010 on JGI IMG/m platform (http://img.jgi.doe.gov/cgi-bin/m/main.cgi). The next step is to resolve the gaps within scaffolds. Typically, PCR reactions targeting specific gaps are run and then the resulting amplicons are sequenced. Instead, we used a different approach that resolved assembly gaps *in silico* using existing sequencing data. Assuming a gap was not caused by insufficient sequencing coverage, we searched with BLASTN against all contigs assembled by Newbler for overlapping contigs that could be candidates to bridge the gap (Figure 2a). A perl script (Text S1) was composed to automate the searching process. Briefly, this program begins by retrieving 1000 bp sequences from the edges of two presumed neighboring contigs. Using BLASTN (typically DNA sequence identity >95% and e-value <1e−10) searching against all contigs assembled by Newbler, other contigs that overlaps nicely with these two 1000 bp sequences were identified. Imperfect sequence overlapping (<100% DNA sequence identity) is allowed considering potential variations caused by subspecies polymorphism and repetitive elements that Newbler cannot resolve properly. From the 5′ side of the gap, each potentially overlapping contig identified was used to repeat the search in the next iteration. After each iteration, the new overlapping contigs were compared to those identified from a similar process initiated from the 3′ edge of the assumed gap. If a common overlapping contig was identified from both ends, a potential solution was suggested and output. A typical output of this program is shown in Figure 2b. These overlapping contigs identified by the perl program were then input into a sequence manager, Geneious Pro v. 5.4 [16], for sequence alignment and further analysis (Figure 2c).

Using this approach, we were able to resolve nearly all the gaps caused by repetitive elements and provide alternative solutions to gaps caused by subspecies polymorphism. Moreover, by attempting to close potential gaps between the terminal contigs of scaffolds, we were able to determine if any two scaffolds were adjacent, and if so, we could provide solutions to the gaps between them just like to a gap within a scaffold. In this way, we successfully determined the order of *Dehalobacter* scaffolds and combined them into a closed circle. However, this circle turned out to be a chimeric genome, a combination of two *Dehalobacter* genomes coexisting in the ACT-3 metagenome: in many gaps, the solutions consisted of alternative contigs that belong to the genomes of the two co-exisiting *Dehalobacter* strains.

### Separation of the Two *Dehalobacter* Genomes

**Figure 3:**
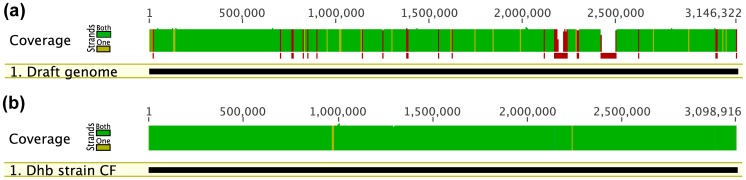
Separation of the genome of strain CF50 by progressive read-mapping. (a) the result of 1^st^ read mapping against the draft reference genome. (b) The result of last read mapping against the refined reference genome. Illumina read pairs from the CF metagenome, which only has the genome of strain CF50, were mapped against a reference genome derived from a chimeric *Dehalobacter* genome from the ACT-3 metagenome, which has both strain CF50 and strain 11DCA. The progressive read-mapping process as described resulted in the refined genome (Figure 2b), representing the genome of strain CF50. Regions that have coverage lower than 5x are highlighted in red. The read depth is highlighted in green when both DNA strands were covered and in yellow when only one strand was covered.

Separation of these two *Dehalobacter* genomes using sequencing data from the ACT-3 metagenome alone was impossible. Fortunately, we had Illumina read pairs from the CF metagenome, which contained only one *Dehalobacter* strain. The two genomes were separated by mapping the Illumina reads from the CF metagenome against the chimeric genome from the ACT-3 metagenome. First, a reference genome was created from the chimeric genome by using the consensus sequence in regions where the two genomes differed because of SNPs. In regions where the two genomes differ dramatically, we screened alternative sequences until we found the one that agreed with the Illumina data. Geneious Pro offers a powerful read-mapping program that allows mapping only with read pairs; this imposes extremely high read-mapping accuracy. After the first read mapping process, regions with poor coverage (read depth<5) were identified (Figure 3a); these regions were mostly where the wrong alternative sequences were chosen and incorporated into the reference genome. By switching alternative choices in these regions, the reference genome was refined (Figure 3b).

Another function of this read mapping process was to verify the solutions to gaps proposed by the *in silico* gap resolution process. Often, if the wrong or false solution was selected, it would result in a region of poor coverage (except those regions caused by subspecies polymorphism described above). However, not all false solutions could be identified by mapping with Illumina read pairs. One example is in the case of tandem repeats, which is discussed below in “Results”. Another example is related to transposable elements that tend to cause sequence duplication at their target sites. The duplicate sequences (often located on both sides of a transposable element), especially those longer than the length of 454 reads, can lead to a false solution that favors the deletion of the transposase gene. Such a false solution cannot be detected by read mapping. To avoid this pitfall, for gaps that would allow both the insertion or deletion of a multi-copy sequence (often a transposase gene), we always first tested the option including the insertion first, when mapping with Illumina read pairs.

After this trial-and-error process of progressive read mapping, the *Dehalobacter* genome shared by both metagenomes was identified (Figure 3b); the genome was named *Dehalobacter* sp. strain CF50. By manually filtering the alternative gap solutions of the chimeric genome from the ACT-3 metagenome against the genome of strain CF50, the other *Dehalobacter* genome, named *Dehalobacter* sp. strain DCA, was also assembled.

### PCR Reactions

There were still four gaps that could not be fully resolved *in silico*: three of them were located within the three ribosomal RNA operons in both *Dehalobacter* genomes, and the other one was a large repetitive region (∼800 bp long) consisting of continuous repetition of oligonucleotides. The complete resolution of these four gaps was achieved by PCR amplifications followed by Sanger sequencing targeting uncertain regions on the amplicons. In addition, to verify our *in silico* gap resolution approach, PCR primers were designed to 22 gaps in the genome of strain CF50. These gaps were caused by the presence of repeats but resolved completely *in silico*. In all PCR reactions, DNA from the CF subculture, containing only strain CF50, was used as the template. Primer design was facilitated by the primer design function offered in Geneious Pro. For long-distance amplifications (>3 kb), Phire® Hot Start II DNA polymerase (Fermentas, Canada) was used; in other cases, Taq DNA polymerase (New England Biolabs, Canada) was used. The temperature programs were designed based on the properties of the primers and the instruction manuals for the two enzymes. The size of amplicons was estimated by electrophoresis on a 1% agarose gel and comparing bands to those from a DNA ladder (GeneRuler™ 1 kb Plus; Fermentas, CA). Direct Sanger sequencing of the amplicons was performed by the Centre of Applied Genomics (Toronto, Canada).

### Genome Polishing

The two *Dehalobacter* genomes were polished further mainly with read mapping and editing in Geneious Pro. The genome of strain CF50 underwent two polishing steps. First, we mapped short-insert (∼647 bp) Illumina read pairs from the CF metagenome against the CF50 genome to correct errors caused by the inaccuracy of 454 pyrosequencing in estimating the length of homo-polynucleotides. The mapped Illumina read pairs generated a polished genome with limited SNPs (defined as positions that have a variant frequency higher than 10%). These SNPs were mainly caused by polymorphisms among multi-copy sequences within the genome. They were located so deeply inside multi-copy sequences that they could not be fixed by short-insert read pairs. Second, we corrected these SNPs by mapping with long-insert (∼8 kb) 454 read pairs from the ACT-3 metagenome.

Since the genome of strain DCA was only present in the ACT-3 metagenome and there is no Illumina paired-end data that can be used to polish it as for the genome of strain CF, it was polished slightly differently. First, a concatenated sequence containing the draft genome of strain DCA with the polished genome of strain CF50 was created using a 20 kb long poly-N bridge which is longer than any read pair span. Against this sequence, we mapped all 454 reads (either in pairs or not) and identified the SNPs that were not related to homo-polynucleotides but had a variant frequency higher than 10%. Nearly all of these SNPs were caused by two nucleotides (40% A and 60% T). For those consisting of more than 90% of one nucleotide, this nucleotide was chosen. Others were assumed to be caused by subspecies polymorphism. The positions of these SNPs were marked and the genome of strain DCA was aligned with that of Strain CF50. For the marked SNPs, if one nucleotide was used by strain CF50, the other nucleotide was chosen for strain DCA. For variations between the two genomes that were related to sequences of homo-polynucleotides, we refined the genome of strain DCA by harmonizing it to that of strain CF50. Notably, because the genome of strain DCA could not be polished directly using read mapping of Illumina paired-end data, potential sequence errors intrinsic to 454 pyrosequencing cannot be fully corrected for this genome. However, we expect these errors to be minimal, because most errors caused by 454 pyrosequencing appeared as homopolynucleotides based on our observations during the polishing of the genome of strain CF and given the extreme similarity of the two strains, most of these errors would have been corrected using the genome of strain CF as the reference.

### Testing on a Published Genome

To further validate the approach, we attempted to re-assemble a published genome of *B. salanitronis* [8]. The raw sequencing data of the genome was kindly provided by the JGI. The data consists of two sets of sequencing data derived from pure culture DNA: one set was generated using 454 pyrosequencing, which included both mate-pair (average insert size of 6465±1616 bp) and non-mate-pair sequence data; and the other set was generated using Illumina technology and provided non-mate-pair (average read length of 36 bp) sequence data. The 454 data was first assembled with Newbler v. 2.3 accessed through Galaxy JGI (https://galaxy.jgi-psf.org/) and default settings were used. The draft assembly with Newbler produced 121 contigs in one scaffold; other scaffolds that belong to plasmids were not considered. Our *in silico* gap resolution strategy was then applied to these contigs, resulting in a closed genome. We could not verify the assembly using Illumina data as we did for the genome of *Dehalobacter* strain CF50 because the data was not in pairs. Instead, we mapped the 454 long-insert read pairs against the assembled genome. We further polished the genome with Illumina reads to correct sequencing errors in homopolynucleotides in the 454 sequence data. We then mapped 454 paired reads and single reads to further correct ambiguities.

### Accession Number

The sequences and annotations of the two *Dehalobacter* genomes have been deposited in NCBI with the following accession numbers: CP003870 for strain CF and CP003869 for strain DCA.

## Results

### Draft Assembly of the ACT-3 Metagenome

**Figure 4:**
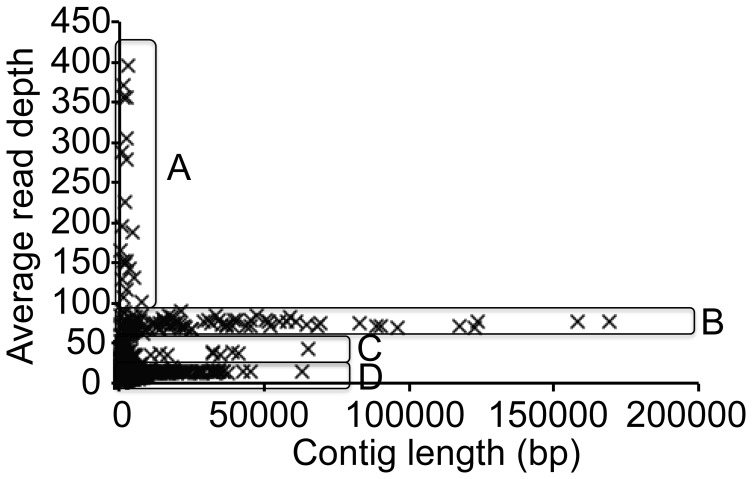
Contig distribution in the ACT-3 metagenome. Based on average read depth, the contigs were grouped into 4 regions. Region A: multi-copy contigs in the *Dehalobacter* genomes (read depth>90); Region B: contigs shared by both *Dehalobacter* strains (red depth ∼70); Region C: contigs specific to each *Dehalobacter* strain (red depth ∼35); Region D: contigs that belong to other organisms of lower abundance (red depth<20).

454 pyrosequencing of the ACT-3 culture generated ∼2.2 M reads with the average read length of 198 bp. Approximately 0.9 M reads were in pairs with an insert size of ∼8.6 kb. The draft assembly of the ACT-3 metagenome generated 28,621 contigs and 331,559 singlets, which can be accessed through IMG/M (http://img.jgi.doe.gov/cgi-bin/m/main.cgi) with IMG taxon object ID, 2100351010. There were 13,437 contigs longer than 500 bp, with N50 of 1705 bp. The largest contig was 169,374 bp long. As the read depth of a contig is proportional to its abundance, these contigs were classified according to read depth (Figure 4). Subsequent assembly proved the coexistence of two *Dehalobacter* genomes in the ACT-3 metagenome. Therefore, there were contigs shared by both *Dehalobacter* genomes with read depth of ∼70 (Region B in Figure 4) and there were contigs specific to each of the two *Dehalobacter* genomes with read depth of ∼35 (Region C in Figure 4). Contigs with read depth higher than 90 (Region A in Figure 4) mainly belong to multi-copy sequences (such as transposable elements and ribosomal RNA sequences) in *Dehalobacter* genomes, while contigs with read depth lower than 20 (Region D in Figure 4) belong to less abundant organisms in the ACT-3 culture. The most abundant (non-*Dehalobacter*) fermenting organism in the ACT-3 metagenome was a strain of *Bacteroides*, with read depth of ∼14. Many of these contigs were further combined into scaffolds by Newbler incorporating mate-pair constraints. Overall, 159 scaffolds were generated. The largest scaffold had an estimated length of ∼2.7 Mb. Ironically, this scaffold did not belong to *Dehalobacter*, but to *Bacteroides*. In order to assemble the genomes of *Dehalobacter*, we identified *Dehalobacter* scaffolds as those with read depth higher than 20 (Table 1).

**Table 1:** *Dehalobacter* scaffolds in the ACT-3 metagenome (scaffolds of other organisms are not included in this table).

Scaffolds	From	To	No. of Gaps
Scaffold002	Contig00228	Contig00268	40
Scaffold003	Contig00269	Contig00297	28
Scaffold004	Contig00298	Contig00314	16
Scaffold009	Contig00530	Contig00539	9
Scaffold018	Contig00677	Contig00678	1
Scaffold041	Contig00883	Contig00885	2
Scaffold054	Contig00972	Contig00975	3
Scaffold095	Contig01153	Contig01154	1
Scaffold129	Contig01244	Contig01245	1

### *In Silico* Gap Resolution

There were 101 gaps within 9 *Dehalobacter* scaffolds (Table 1). Using *in silico* gap resolution (Figure 2), we were able to close almost all these gaps from remaining contigs pre-assembled by Newbler. The gaps were classified by type into four Groups (A, B, C and D). We adopted a gap nomenclature based on neighboring contigs: for example, the gap between contig00290 and contig00291 was written as 00290-G-00291.

#### Group A (overalapping contigs, 23 gaps)

**Figure 5:**
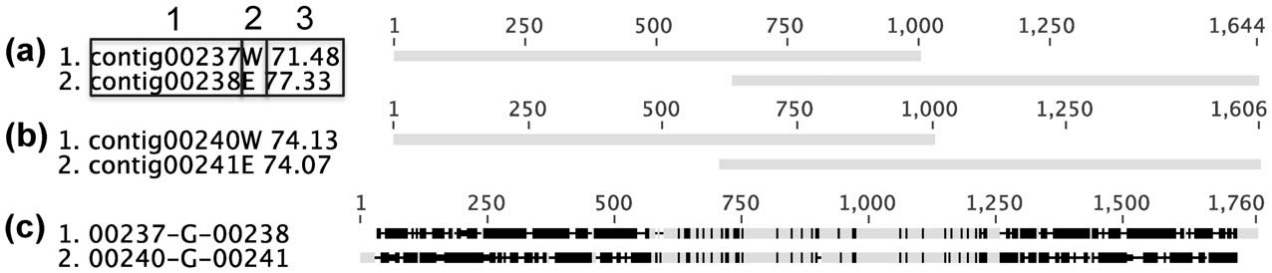
Typical gaps in Group A. (a) The resolution of gap 00237-G-00238. (b) The resolution of gap 00240-G-00241. (c) The sequence alignment of the consensus sequences of gap 00237-G-00238 and gap 00240-G-00241. All DNA sequence alignments (including those in other figures) were generated with Geneious Pro, having the same format. As shown in Figure 5a, most sequence identifiers consist of three regions. Region 1 shows the ID of the sequence. Region 2 indicates some specific tags: “W” means the sequence is the last 1000 bp nucleotides adapted from the 3′ end of the contig, and it is on the west side of the gap; “E” means the sequence is the first 1000 bp adapted from the 5′ end of the contig, and it is on the east of the gap; “F” means the sequence is a whole contig and in its forward orientation; “R” means the sequence is a whole contig but in its reverse orientation. Region 3 shows the average read depth of the contig from which the sequence is derived. The sequence alignment is shown on the right hand side. Marks on the top show the scale; the alignment mismatches are highlighted in black and the matches in grey; gaps in sequences are indicated in dashes. In some Figures (e.g., Figures 7, 10, 14) the identity of the overlapping sequences is shown on top of the alignment as a coloured bar; positions with 100% identity are in green and positions with lower identity are in yellow.

For gaps in this group, the preceeding contig overlapped directly with the following contig, yet were not assembled. Many of these gaps were caused by the presence of a repeated sequence of 500–700 bp present in two copies in the genome (Figure 5). The two copies were not necessarily identical, but contained homologous regions that broke the assembly. These kind of repeated sequences tend to exist in pairs: 00237-G-00238 with 00240-G-00241, 00252-G-00253 with 00286-G-00287, 00256-G-00257 with 00261-G-00262, 00257-G-00258 with 00260-G-00261, 00280-G-00281 with 00281-G-00282, 00294-G-00295 with 00295-G-00296, and 00300-G-00301 with 00301-G-00302. Examples of the sequence annotations in these gaps include: export cytoplasm protein SecA, ATPase RNA helicase, invasion associated protein p60, spore germination B3 GerAC, flagellin protein FlaA and hypothetical proteins. The two homologous copies of each gene are probably paralogs in the genome. For the remaining 7 gaps in Group A, the reason why they were not assembled is unknown; three of these 7 gaps were chosen for verification with PCR and subsequent sequencing of the amplicons: these additional sequencing results agreed with those determined by our *in silico* gap-resolution method (Table S2).

#### Group B (multi-copy contigs, 38 gaps)

**Figure 6:**
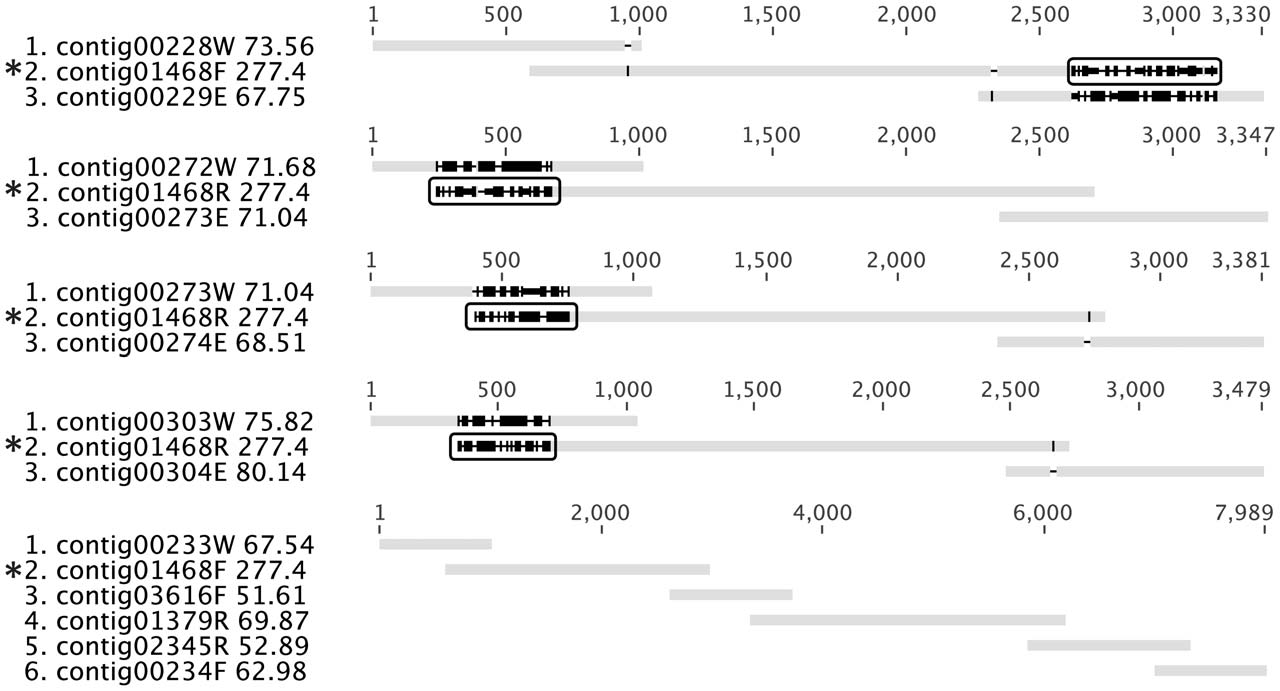
Typical gaps in Group B. Five gaps caused by the presence of a multi-copy contig, contig01468 are shown. Notably, although part of contig01468 is shared by all five gaps, the terminal part on the 5′ edge of contig01468F (highlighted with rectangles) only belongs in the last gap. It would be more reasonable to assemble the raw reads in this region to contig03616, but Newbler was not smart enough to do so. The consequence is that this kind of poor overlap (as shown in the first four gaps) prevailed in the resolution of gaps caused by multi-copy contigs. Accordingly, these poorly overlapping edges of the multi-copy contigs were trimmed in the construction of consensus solutions.

Gaps in this group could often be filled by the placement of contigs that have high read depth and thus exist in multiple copies in the genome. Most of these multi-copy contigs contained sequences for repetitive elements commonly found in bacterial genomes (Table 2), including the genes of transposases, reverse transcriptases, integrases, and ribosomal RNAs. In the resolution of these gaps, the overlap between these multi-copy contigs and other contigs was not necessarily perfect (Figure 6). A multi-copy contig is originally assembled from reads that come from different loci of the genome, so it is prone to be chimeric. Therefore, the multi-copy contig can have an edge sequence that is specific to one of its multiple loci on the genome, causing an imperfect overlap in other loci (Figure 6). The poorly-overlapping edge from the multi-copy contig therefore must be trimmed in resolving these gaps (rectangular boxes in Figure 6).

**Table 2:** Length, read depth and annotation of multi-copy sequences in the *Dehalobacter* genomes.

Contig ID	Length (bp)	Average Read Depth^1^	Annotation
01677	1957	75.4	Putative transposase
06868	738	90.0	–
02118	1533	106.8	PBS lyase HEAT-like repeat family protein
01493	2317	115.7	Putative reverse transcriptase
05122	872	128.7	*Dehalobacter* 16S rRNA (partial)
01334	3439	142.1	Putative recombinase
01997	1660	149.0	*Dehalobacter* 16S rRNA (partial)
02840	1270	151.2	Putative transposase
01481	2332	151.2	Putative transposase
01315	4355	187.9	*Dehalobacter* 5S and 23S rRNA
04728	914	194.78	Putative transposase
01581	2052	226.0	Putative transposase
01468	2361	277.4	Putative transposase
04522	940	287.5	Putative transposase
01532	2202	304.1	Putative reverse transcriptase
03012	1216	355.2	Putative transposase
01504	2287	355.6	Putative transposase
02363	1449	371.5	Putative transposase
01388	2750	395.1	Putative transposase

1 The average read depth of the contigs shared by both *Dehalobacter* genomes is 69.5.

#### Group C (strain variation and alleles, 16 gaps)

**Figure 7:**
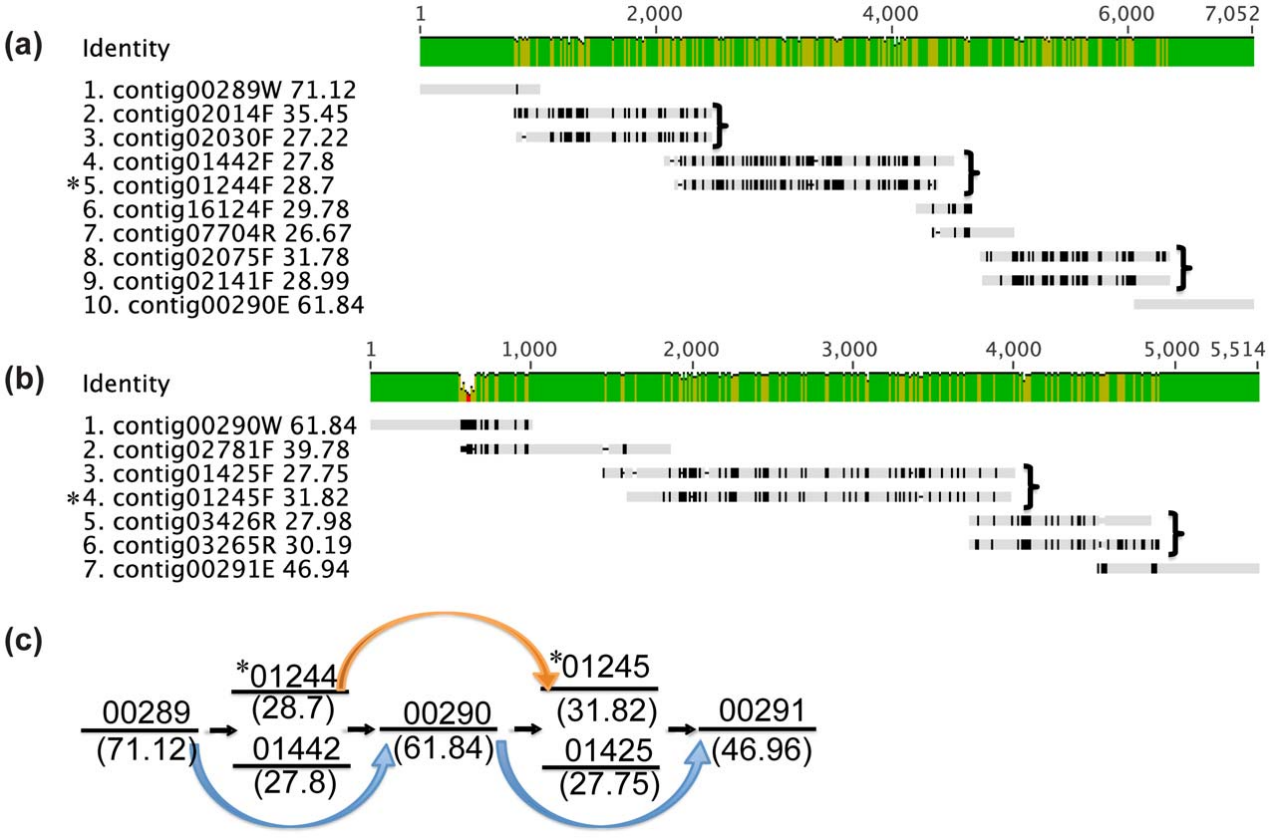
Typical gaps in Group C. (a) The resolution of gap 00289-G-00290. (b) The resolution of gap 00290-G-00291. In Figure 7a and 7b, “pairs of alternative contigs” are highlighted in single brackets; contig01244 and contig01245 are highlighted with an asterisk. (c) The schematic graph showing the relationship between scaffold003 and scaffold129. Contigs are represented by straight lines with contig ID on the top and average read depth at the bottom; curved arrows indicate scaffolding relationships.

Subspecies polymorphism (or strain variation) is a challenge specific to metagenomic data. The 21 gaps in Group C resulted from differences between the two *Dehalobacter* genomes coexisting in the ACT-3 metagenome. A distinctive feature of all gaps of Group C is the presence of “pairs of alternative contigs” (Figure 7a and 7b); the number of the pairs of alternative contigs varies for different gaps. The two alternative contigs are homologous and have limited differences, often in the form of dispersed SNPs. They also tend to have similar length and similar read depth (Figure 7a and 7b). The presence of these “pairs of alternative contigs” further confirms the presence of two *Dehalobacter* genomes in the ACT-3 metagenome.

#### Group D (insertions and deletions, 23 gaps)

**Figure 8:**
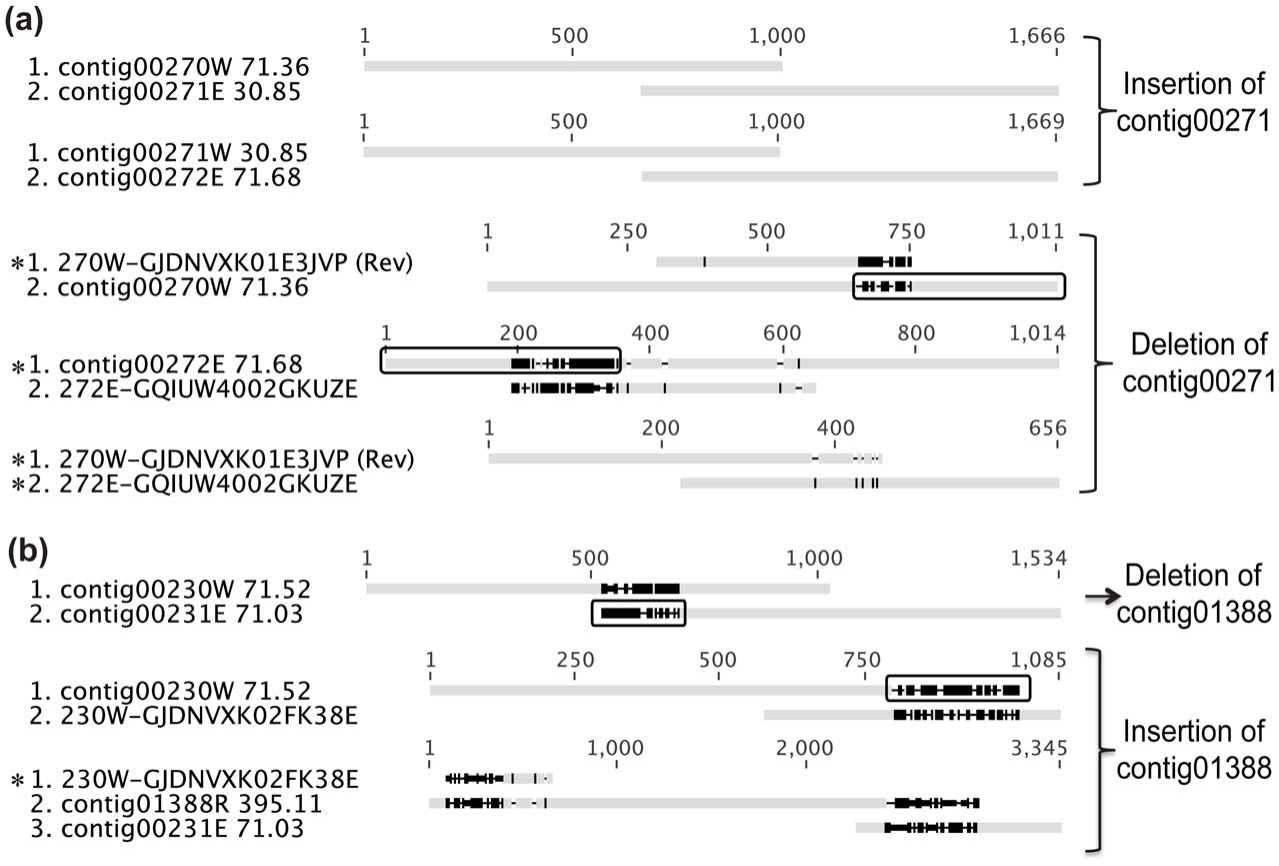
Typical gaps in Group D. (a) The insertion or deletion of contig00271. (b) The insertion or deletion of contig01388. The sequences highlighted with an asterisk are raw reads that are suppressed at the edges of different contigs. Sequence edges that are highlighted in rectangles should be trimmed in generating consensus sequences.

Gaps in this group were caused by subspecies polymorphism resulting from the insertion of a sequence in one strain but not in the other. One example is shown in the resolution of three related gaps (Figure 8a): 00270-G-00271, 00271-G-00272, and 00270-G-00272. These three gaps were caused by the fact that contig00271 was present in only one of the two strains. Read depth further confirmed this solution: the read depth of contig00271 is 30.86, while the read depths of contig00270 and contig00272 are 71.36 and 71.68, respectively (Figure 8a), therefore contig00271 only belongs to one strain. Unfortunately, superficial analysis based on scaffolding information of these contigs only favors the insertion of contig00271: these three contigs were in sequential order in scaffold002 and the overlap between contig00270 and contig00271 and between contig00271 and contig00272 appears perfect. The traces that support the deletion of contig00271 were buried in the assembly of related 454 contigs (Figure S1): a phenomenon we named “edge sequence suppression”. These traces can be revealed in the visualization of the assembly at the 5′ edge of contig00270 as shown in Figure S1, which was generated using EagleView v.2.0 [17] where the sequences that were suppressed are highlighted in red. For contig00270, there were 15 homogeneous reads suppressed, represented by the read labeled GJDNVXK01E3JVP (Figure 8a); these suppressed reads constitute an alternative ending to the contig. On the 3′ edge of contig00272, an alternative ending represented by the raw read GQIUW4002GKUZE (Figure 8a) was found. The alignment of these two alternative endings supports the deletion of contig00271 (Figure 8a). Many other similar examples were found (Table S1).

The two alternative paths between contig00270 and contig00272 arose because contig00271 is present in one of the two strains. Contig00271 is only 1148 bp, encoding a gene annotated as “Stage 0 sporulation two-component response regulator”. A similar case was found between contig00253 (read depth of 73.4) and contig00255 (read depth 81.9); in this case, the ambiguity was caused by contig00254 (read depth of 43.1), that is ∼65 kb long and includes 65 genes. In these two cases, the two sequences that were either inserted or deleted were not commonly found as transposable elements. However, in all other gaps in Group D, the insertion or deletion corresponded to a common transposable element. For example, for gap 00230-G-00231 (Figure 8b), the ambiguity was caused by a multi-copy contig, contig01388, which had an average read depth of 395. As discussed earlier, the gaps in Group B were also caused by such multi-copy contigs. The difference between them is that solutions to the gaps in Group B were shared by both *Dehalobacter* strains, while those to the gaps in Group D were strain-specific. It is likely that the transposition events that cause gaps in Group B happened before the differentiation of the two strains, while the transposition events that cause gaps in Group D happened after the differentiation.

#### Gap-distance comparison

**Figure 9:**
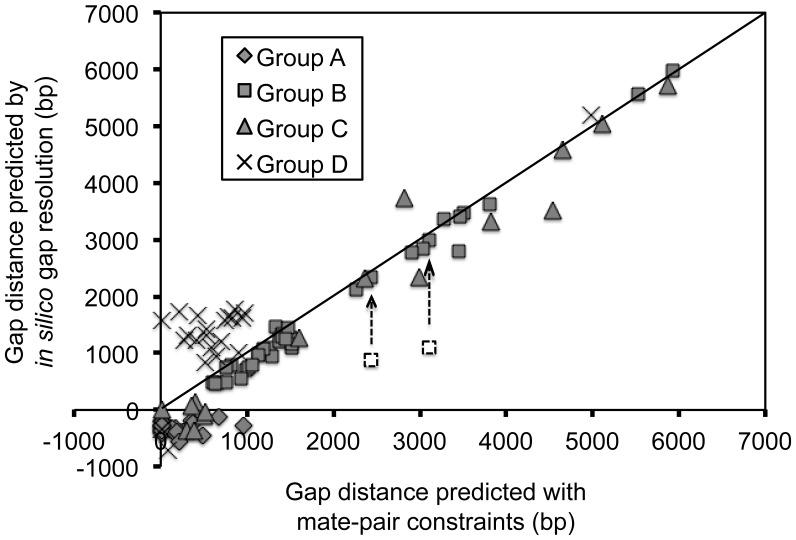
Assessment of the assembly using gap-distance comparisons. When the preceeding contig and the succeeding contig overlapped directly with each other, the gap distance was negative with the value equal to the length of the overlapped region. However, all gap distances calculated from Newbler were positive and the minimum value was 20 (the details of Newbler’s calculation are unknown). This explains why some gaps locate below the horizontal axis. Most gaps from Group D have insertion or deletion of a multi-copy sequence: insertion in one strain and deletion in the other strain. The gap distance based on insertion is longer than the one based on deletion. For simplicity, we calculated gap distance assuming insertion, while Newbler’s estimations should be average values between the gap distance in the case of deletion and the one in the case of insertion, depending on the mate pairs used for calculation. This likely explains why most gaps from Group D locate above the diagonal line. The gap distances for gaps 00285-G-00286 and 00239-G-00240 (highlighted by arrows) are consistent with mate-pair predictions if one assumes the existence of the tandem copies of the multi-copy sequences involved.

To further verify the proposed solutions for gaps within scaffolds, the gap distance determined for the proposed solution was plotted against the gap distance estimated from mate-pair constraints. The latter is obtained from the scaffolding outputs of Newbler and depends on the size distribution of the insert library. Excellent agreement between the two estimates of gap distance was found for most gaps in Group A, B and C (Figure 9). For gaps caused by the insertion or deletion of a sequence (Group D), the gap distance was calculated assuming insertion, while the gap distance determined by Newbler from mate-pair constraints is likely an average of the two cases. This can explain why the mate-pair estimations of the gaps in Group D were found to be lower than those predicted by assuming the case of an insertion (Figure 9).

### Alternative Scaffolds

**Figure 10:**
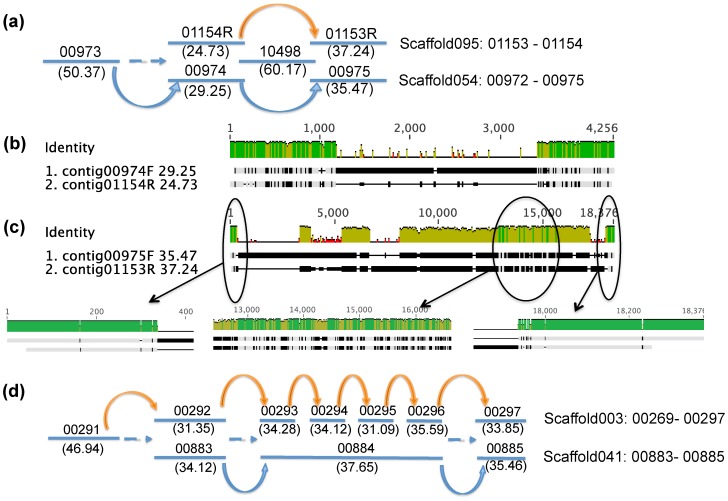
Combinations of alternative scaffolds. (a) The combination of scaffold095 and scaffold054. (b) Traces of homology between contig00974 and contig01154R. (c) Traces of homology between contig00975 and contig01153R. (d) The combination of scaffold041 and scaffold003. Contigs are represented by straight lines with contig ID on the top and average read depth at the bottom; curved arrows indicate scaffolding relationships.

In the discussion of gaps caused by subspecies polymorphism in Group C, we explained the existence of “pairs of alternative contigs”. In those cases, contigs are alternative to each other. In some cases, the whole scaffold becomes the alternative. One simple example is scaffold129, which consists of two contigs: contig01244 and contig01245. Contig01244 is alternative to contig01442 in gap 00289-G-00290, while contig01245 is alternative to contig01425 in gap 00290-G-00291 (Figure 7). Since the two gaps are neighboring, scaffold129 can be incorporated into the two gaps of scaffold003 (Figure 7c). Another example of alternative scaffolds is scaffold095, consisting of contig01153 and contig01154. We found that gap 00974-G-00975 and gap 01153-G-01154 share the same bridging contig, contig10498 (Figure 10a); in addition, contig00973 can be bridged to either contig00974 or contig01154R (R means reverse orientation) (Figure 10a). Moreover, the read depth values of contig01153, contig01154, contig00974 and contig00975 were all around 30, which means they belong to only one strain; however, contig00973 has a read depth of 50.37. The alignment of contig00974 with contig01154R (Figure 10b) and the alignment of contig00975 with contig01153R (Figure 10c) further revealed traces of conservation. Based on these observations, it can be concluded that scaffold095 is alternative to contig00974 and contig00975, located at the 3′ edge of scaffold054 (Figure 10a). Another more complicated example is scaffold041, which consists of three contigs: contig00883, contig00884 and contig00885. It was found that scaffold041 was alternative to contigs located at the 3′ edge of scaffold003 (Figure 10d).

### Order of Scaffolds

**Figure 11:**
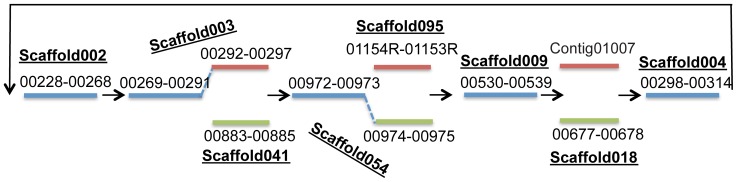
Schematic of the draft chimeric *Dehalobacter* genome from the ACT-3 metagenome. The major scaffolds and contigs are represented as straight lines with contig and scaffold IDs labeled; contigs shared by both strains are in blue; contigs specific to strain CF50 are in read; contigs specific to strain DCA are in green.

Since the 3 alternative scaffolds (scaffold129, scaffold095 and scaffold041) can be incorporated into other scaffolds as described above, there were only 6 scaffolds left. Traditionally, to determine the order of these scaffold and sequences that fill the gaps between them, PCR reactions with primers chosen from the edges of scaffolds are required. Instead, by assuming gaps between any two scaffolds, we resolved the order of scaffolds and the gaps between scaffolds using the same gap-resolution process as for the gaps within scaffolds. Moreover, it was found that scaffold018 (with a read depth of ∼37) is also a strain-specific scaffold. It is an alternative to contig01007, which is 32,699 bp long and has a read depth of 38.73. Using this progressive resolution of gaps within, and then between scaffolds, the overall *Dehalobacter* genome structure was revealed as shown (Figure 11), which is a joint representation of the two *Dehalobacter* genomes. Further polishing was achieved by mapping raw reads back to this assembly.

### Read Mapping

Illumina sequencing of the CF subculture generated ∼27 million mate-pair reads with read length of 76 bp and average insert size of ∼647 bp. In this study, these raw sequencing reads were only used for the separation and polishing of the two *Dehalobacter* genomes by read mapping. The Illumina sequencing data provided average coverage of more than 500x for the genome of strain CF50. The process of read mapping was a vital component of the assembly strategy. It enabled the verification of proposed gap solutions, the separation of the two genomes, and genome polishing. Perfect agreement between raw reads and the reference genome of strain CF50 (Figure 3b) demonstrated the accuracy and effectiveness of our gap-resolution strategy. The separation of the two *Dehalobacter* genomes was achieved by successively mapping the Illumina data from the CF subculture as described in “Materials and Methods”. For genome polishing, the genome of strain CF50 was first polished by mapping short-insert (∼647 bp) Illumina reads pairs, followed by mapping long-insert (∼8.6 kb) 454 read pairs. It was found that mapping with short-insert Illumina read pairs could not resolve all ambiguities, especially those derived from sequence variations among multiple copies of a multi-copy sequence. These recalcitrant ambiguities were then fixed by mapping long-insert 454 read pairs, proving the importance of long-insert mate-pair data in genome polishing.

### Recalcitrant Gaps

**Figure 12:**
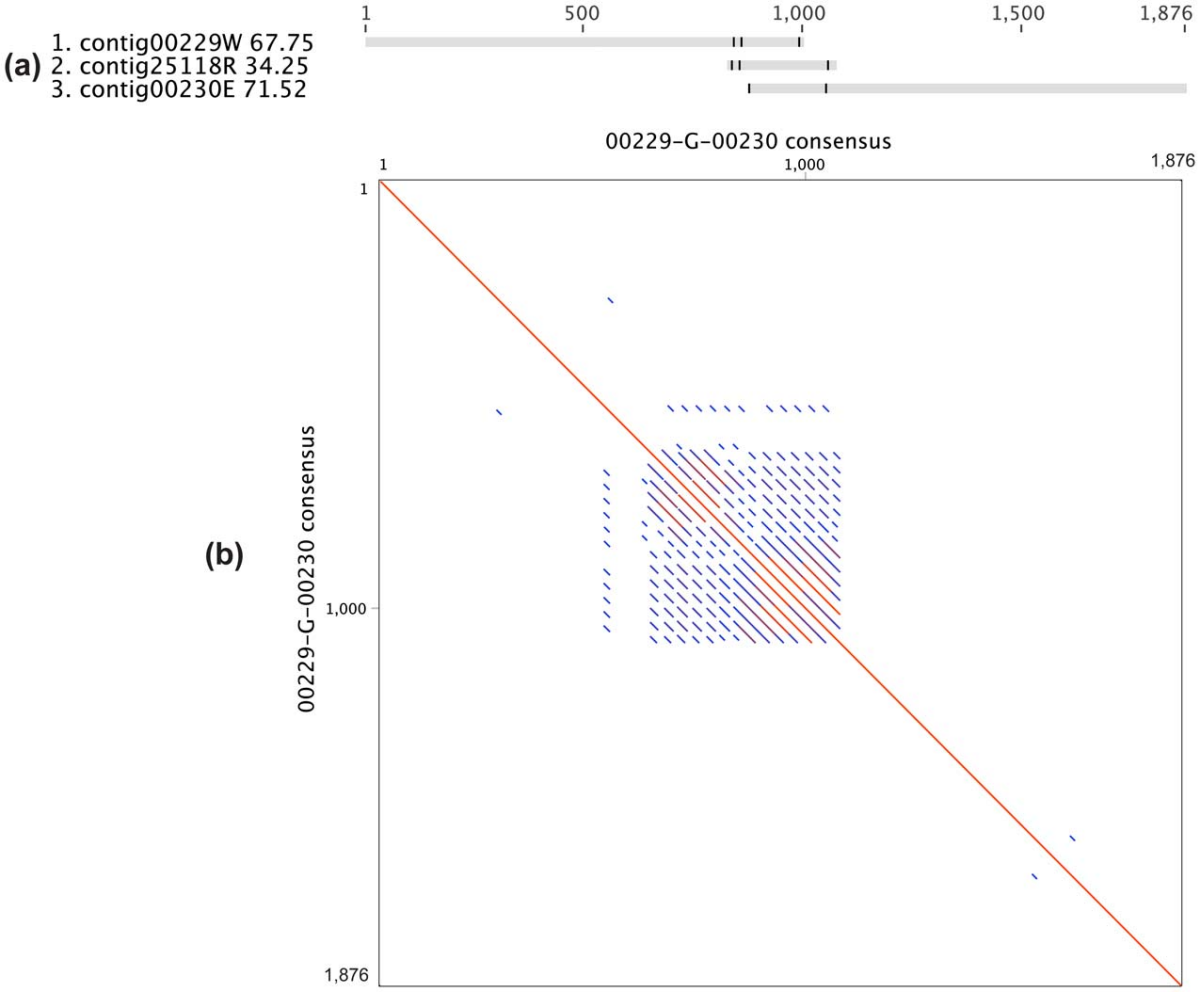
Gap 00229-G-00230. (a) The sequence alignment of related contigs. (b) The dot plot of the consensus sequence from (a) against itself.

Even after the process of read mapping, assemblies in some regions remained problematic. Here are three such examples. The first example is gap 00229-G-00230, which was caused by a continuous repetition of oligonucleotides (Figure 12). Although the presence of such a self-repeated sequence in this gap can be concluded, the size of the repetitive region (>450 bp) can not be determined with 454 or Illumina data. The only way to completely resolve this type of gap is to have a read long enough to cover the whole repetitive region. This gap was eventually resolved by additional PCR amplification and Sanger sequencing.

The second example is two gaps caused by tandem copies of multi-copy sequences: 00239-G-00240 and 00285-G-00286. Mapping with read pairs is very effective to determine if a sequence is present in a gap; however, it is ineffective for determining if the sequence exists in more than one copy in the gap. To determine if such repetitive elements exist in tandem copies, a simple test can be performed *in silico* (Figure S2): two copies of a repetitive element in the same orientation were concatenated with a piece of poly-N (50 bp) in between; then Illumina read pairs were mapped against this artificial sequence (Figure S2). If read pairs spanning the region of polyN can be recovered with correct distances, this repeat must exist in the tandem copies somewhere in the genome. In this way, we identified two repetitive elements that existed in tandem copies: they are the transposable genes related to two multi-copy contigs, contig01504 and contig01532 (Table 2). By careful investigation of these two contigs, we recovered the real sequences that covered the polyN region connecting the tandem copies. Coincidently, we found that two gaps caused by these two multi-copy contigs, 00239-G-00240 and 00285-G-00286, had their gap distances underestimated initially (Figure 9). For example, if one single copy of contig01504 existed in the gap of 00239-G-00240, the gap distance should be 1106 bp, which is significantly smaller than the gap distance predicted from mate-pair constraints, 3111 bp. But if two tandem copies are there, the gap distance becomes 3004 bp, which is consistent with the predicted length. Based on these observations, we concluded the existence of tandem repeats in gap 00239-G-00240 and gap 00285-G-00286. This conclusion has been further confirmed with additional PCR reactions (Table S1).

**Figure 13:**
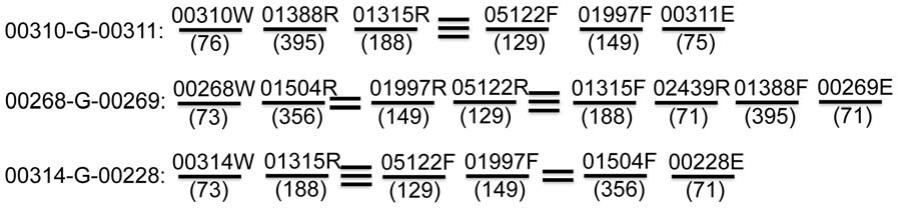
The incomplete resolution of three gaps in which 5S, 16S, and 23S rRNA genes locate. Straight lines represent contigs with contig IDs (top) and average read depth (bottom) indicated. The three lines between contig01315 and contig05122 indicate three potential connections between them; and the two lines between contig01504 and contig01997 indicate two potential connections.

The last example is three gaps caused by the complicated combination of multi-copy contigs: 00310-G-00311, 00314-G-00228 and 00268-G-00269. Gap 00310-G-00311 is within scaffold004; 00314-G-00228 connects scaffold004 and scaffold002; 00268-G-00269 connects scaffold002 and scaffold003. The complicated scenarios of these three gaps stem from the fact that each gap harbored a ribosomal RNA operon, which contains one copy of each of the 5S, 16S and 23S ribosomal RNA genes. While current *in silico* gap resolution revealed the overall structure (Figure 13), ambiguities remained in 01315-G-05122 and 01997-G-01504 (Figure 13). Three options were found to close 01315-G-05122, while two options were found for 01997-G-01504. Interestingly, these multiple options to resolve the gaps did not stem from species polymorphism but variations related to multi-copy sequences within the genome. Such ambiguities were located between long multi-copy contigs, that is, beyond the unique mapping coverage of short-insert read pairs. Long-insert 454 read pairs have the potential to solve these ambiguities; however, our trials on this direction did not generate satisfactory results, probably due to the limited coverage (∼11x) of 454 long-insert mate-pair data. Finally, we had to turn to long-distance PCR amplifications, followed by sequencing with primers targeting the regions of ambiguities. Notably, although current strategy of *in silico* gap resolution failed to resolve all uncertainties in these three gaps, it uncovered the overall layout correctly, which dramatically simplified additional sequencing efforts.

### PCR Verification

The solutions of 19 gaps (mostly from Groups B and D) caused by the presence of long multi-copy contigs were chosen for further verification with PCR reactions. These gaps were caused at least partially by the presence of one of the three multi-copy contigs: contig01388, contig01504 and contig01532. Positive amplifications with expected amplicon size were achieved in all PCR reactions (Table S2), except those for the two gaps, 00239-G-00240 and 00285-G-00286, in which tandem copies of multi-copy sequence were expected. Surprisingly, for both of these two gaps, two amplicons with correct size corresponding to single copy and tandem copies were amplified simultaneously. Similar PCR reactions were designed to confirm the solutions to three gaps in Group A; amplicons with expected size were also obtained for them (Table S2). The amplicons of all 22 gaps were further sequenced using Sanger sequencing: for 21 gaps of them, Sanger sequencing generated sequences that match the expected amplicons derived from the assembled genome. Gaps caused by subspecies polymorphism were not chosen for PCR verification because we believe the solutions to these gaps had been solidly verified in the process that was used to separate the two *Dehalobacter* genomes: in every position where two alternative choices coexisted in the ACT-3 metagenome, only one of them existed in the CF metagenome.

### The Two *Dehalobacter* Genomes

**Figure 14:**
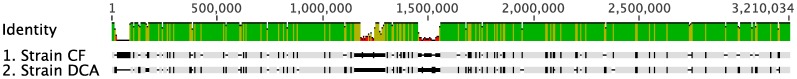
The alignment of the two *Dehalobacter* genomes: strain CF50 and strain DCA.

The finished genome of strain CF50 is 3,092,048 bp long, and the finished genome of strain DCA is 3,069,953 bp long. These two genomes share an identity of 90.6% in DNA sequence (Figure 14). The major differences between the two genomes relate to insertions or deletions of large DNA sequences (such as contig00254, ∼65 kb) and transposable elements, and to alternative contigs or scaffolds (Figure 14). The absence of significant recombination of large genome sequences and the limited amount of SNPs indicating the close relationship of these two *Dehalobacter* genome. These are the first two genomes of *Dehalobacter*. Further analysis and annotation of these two genomes is underway (manuscript in preparation). Two sequences and initial annotations of these two genomes are provided as supplemental files.

### Testing Re-assembly of a Published Genome

**Figure 15:**
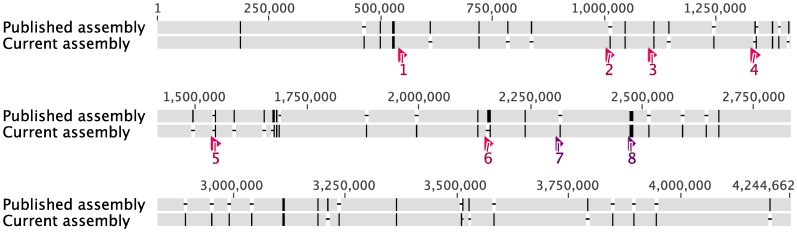
Alignment of the published assembly versus the new (this study) assembly of the *B. salanitronis* genome. The positions of assembly gaps caused by the 6 copies of the rRNA operons are indicated as Region 1–6. Region 7 and 8 indicate the two large regions of disagreement.

To test the applicability of this approach to other genomes or metagenomes, we attempted to assemble the raw sequence data of the published genome of *B. salanitronis* and were able to assemble a genome that agrees well with the previously published genome (Figure 15) that was closed with substantial additional wet lab work. Out of the total length of ∼4.24 Mb, the variations between the two assemblies consisted of 119 SNPs and two insertion/deletion regions. The two insertion/deletion regions are highlighted in Figure 15 and annotated as Region 7 and Region 8. For Region 7, our strategy failed to identify a solution and additional sequencing would be necessary for closure. For Region 8, our strategy suggested the presence of tandem copies of a sequence, while the published assembly has one copy. Read mapping with 454 reads agrees with the existence of two tandem copies (data not shown). Moreover, the existence of the tandem repeats explains why the assembly broke at gap 8. Thus our strategy may actually correct an error in the published genome.

This *Bacteroides* genome has six copies of ribosomal RNA operons resulting in six large assembly gaps (Region 1 to Region 6, Figure 15). The resolution of these gaps was challenging just as what we found for similar gaps in *Dehalobacter* genomes. Although we cannot resolve all ambiguities in these gaps, we managed to uncover the overall layout of bridging contigs, which will simplify additional PCR and sequencing efforts. Many of the ambiguities in these gaps showed up as SNPs when the current assembly is compared to the published assembly. 41 out of 119 SNPs belong here. Of the rest 78 ( = 119–41) SNPs, 31 were caused by disagreements on the length of monopolynucleotides. Re-examination of these 31 SNPs using read mapping with Illumina raw reads showed that we made the correct call for 30 of them and the wrong call for only one, located in a region of low coverage. Therefore, our polishing method resolves errors in mono-polynucleotides more effectively. Re-examination of the rest 47 (78–31) SNPs using read mapping with 454 long-insert read pairs showed that most of the choices we made in these SNPs were supported by the long-insert 454 mate-pair data. As discussed earlier, mapping with long-insert mate-pair data is critical for genome polishing; however, this was not included in the current version of the published assembly. Except the 41 SNPs located in the 6 gaps caused by ribosomal RNA genes, the other 78 SNPs resulted from the different polishing processes used. In the published assembly, the authors used Polisher [18] to polish the assembled genome with Illumina reads [8], while we used read mapping with both Illumina and 454 mate-pair reads.

In summary, the published genome required 193 additional PCR reactions and 4 shatter libraries to close the gaps after the application of GapResolution [8]. Using our approach, we were able to generate a reliable assembly that only requires additional efforts to resolve uncertainties in 7 regions in the genome (Region 1–7, Figure 15). Also, our polishing method appears to have better performance for this genome.

## Discussion

Metagenome sequence assembly is challenging. Satisfactory assemblies have only been reported with sequences from microbial samples from extreme environments [19] or enriched laboratory cultures [20], [21] that tend to have simpler community structure. However, even in these cases, the assemblies suffer from subspecies polymorphism [19], [20]. In this study, the ACT-3 metagenome was dominated by two highly similar *Dehalobacter* strains, resulting in severe fragmentation in the assembly produced by Newbler: subspecies polymorphism factored in about half of the assembly gaps in these *Dehalobacter* genomes. As with most genome assemblers, Newbler cannot address problems caused by subspecies polymorphism.

We propose a straightforward and effective solution that uses pre-assembled contigs to bridge gaps. The incorporation of read mapping for verification significantly increased the robustness and accuracy of the process. This strategy resulted in the *in silico* resolution of nearly all gaps caused by repetitive elements and subspecies polymorphisms in the *de novo* assembly of the two *Dehalobacter* genomes. Its power and general applicability were further demonstrated in the re-assembly of a published *Bacteroides* genome. Using pre-assembled contigs as building blocks, this strategy was not restricted to a particular sequencing technology or sample type (metagenomic or genomic). However, in searching for overlapping contigs, the length of the overlap often depends on sequencing technology and the program used to generate contigs. When Velvet [22] and Allpath [23] are used with Illumina reads, the maximum overlap between contigs is shorter than the K-mer size, which is shorter than the Illumina raw reads (20–150 bp). But in the assembly of short-read mate-pair data, contigs assembled with ABySS [24] tend to have much longer overlap. Our in silico gap-resolution strategy worked well when ABySS was used for contig assembly and SSPACE [25] for scaffolding.

Recent studies address specific challenges in metagenomic assembly. Genovo [10] was designed to differentiate read variations caused by sequencing noise from those caused by true biological variation, which enabled better assembly, especially for the organisms in low abundance. Bambus 2 [11] was designed to overcome challenges in scaffolding caused by subspecies polymorphism. Iverson *et al*. [26] improved metagenomic scaffolding by considering not only mate-pair information, but also nucleotide composition and read-depth distribution. Meta-IDBA [9] proposed to capture the slight variations within closely related strains by multiple sequence alignment and represent the sequence of one species by condensing the alignment. Gaps caused by subspecies polymorphism prevail in metagenomic assembly: in current study, a simple scenario in which only two interfering genomes coexisting was encountered. However, ∼40% of all gaps were related to subspecies polymorphism. Our gap-resolution approach proposed “partial” solutions to these gaps: representing alternative choices in multiple sequence alignment. Obviously, further condensation of such an alignment as occurs with Meta-IBDA [9] risks creating reading frame shifts and introducing more confusion; therefore, multiple sequence alignment appears a better solution to these gaps. Using multiple sequence alignment to resolve the gaps caused by subspecies polymorphism, our gap-resolution approach assembled all *Dehalobacter* contigs in the ACT-3 metagenome (two *Dehalobacter* strains) into a closed assembly. If the CF metagenome data were not available, this closed assembly is still a dramatic improvement over the initial set of contigs and scaffolds assembled by Newbler. Therefore, the proposed gap-resolution approach is not only useful in our special case; rather it has the potential of improving the assembly of other all metagenomic data sets containing two or more interfering or closely related strains. However, to resolve individual genomes and assign alternative sequences in the gaps caused by subspecies polymorphism, additional information was required, such as a genome or metagenome of an individual strain (the CF metagenome in our case). This technique could be particularly useful if genome sequence from DNA amplified from single cells sorted out of a metagenome were available, for example.

Three standalone *in silico* gap-resolution programs, IMAGE [6], GapResolution (http://www.jgi.doe.gov/software/) and GapFiller [7], have been reported previously to improve preliminary assembly of single genomes. Unlike our strategy, these programs close assembly gaps by extending the neighboring contigs using mate-pair raw reads. Therefore, the resolution of a gap using these programs is subject to the coverage of mate-pair data over the gap. Most importantly, being designed to close gaps in the context of a single genome, these two programs cannot resolve gaps that have non-unique solutions, especially those caused by subspecies polymorphism. Our strategy relies on sequence overlapping between contigs to close gaps and then uses mate-pair data and read mapping for assembly verification. The closure of a gap typically requires the alignment of a small number of contigs, but would require thousands of raw reads. Therefore, dealing with contigs is computationally more efficient than with raw reads. While our gap resolution approach has yet to be fully automated (it relies on manual inspection, consideration of read-depth, and analysis and adjustment of the alignment of the overlapping contigs), it can resolve most gaps of a regular bacterial genome *in silico* and accurately. The re-assembly of the published *Bacteroides* Genome was done in a few days. The automation of this gap-resolution process is a focus of further work, but will require iterative user-input in the selection of alternative assemblies.

In this study, we also explored the possibility of assembling complete bacterial genomes using second-generation sequencing data only. With genome polishing and most gap resolution work completed *in silico*, we only failed in the resolution of four recalcitrant gaps. We observed that short reads were not a problem except for those gaps caused by multiple repetitions of a motif (Figure 12). Many sequencing facilities offer mate-pair (or paired-end) sequencing of short-insert (<1 kb) libraries because they are easy to prepare and thus low cost. However, the value of mate-pair sequencing of a long-insert library cannot be overemphasized. Because paired-end read data are used by most scaffolding algorithms [11] the maximum distance that can be overcome will be less than the distance between paired reads. This agrees with our results: all gaps within scaffolds were less than 8 kb (Figure 9). Therefore, to a certain extent, the performance of scaffolding increases with the size of the insert in the mate-pair library. In addition to the benefits in long-distance scaffolding, long-insert mate-pair data are indispensable for accurate genome finishing and polishing.

### Conclusions

*Dehalobacter* are strictly anaerobic, organohalide-respiring bacteria that reductively dechlorinate and detoxify common groundwater contaminants; they are important in the global chlorine cycle and for remediation of contaminated sites. Using a new gap-resolution strategy, the first two genomes of *Dehalobacter* were assembled from the metagenomes of two enrichment cultures. The strategy resolves gaps using pre-assembled contigs followed by verification with read mapping. Designed to make full use of existing sequencing data, this new strategy successfully resolved *in silico* nearly all gaps caused by repetitive sequences and/or subspecies polymorphism; only four additional PCR reactions and amplicon sequencing were required to clarify ambiguities. This strategy can be used to enable the accurate assembly of single genomes and metagenomes, and substantially reduce additional efforts required in the web lab for genome finishing.

## Supporting Information

Figure S1 **Visualization of raw reads suppressed at the 5′ edge of contig00270.** Each row is a raw read. The raw reads that were suppressed (but match each other) are highlighted in red.
(DOCX)

Figure S2 **Detection of tandem repeats by read mapping.** The vertical line in the middle indicates the region of poly-N sequence (50 bp) that is inserted between the tandem copies of the transposase gene related to contig01504. Although the coverage in this region is zero, many read pairs spanning this region were identified, which proves the existence of the tandem copies.
(DOCX)

Table S1 **Contigs that contain reads whose sequences were suppressed.**
(DOCX)

Table S2 **Experimental verification of the resolution of 22 assembly gaps.**
(DOCX)

Text S1 **The perl script that automates the searching of overlapping contigs that can potentially resolve a given gap.** For a specific gap defined by the user (the gap 00310-G-00311 was used for demonstration), BLASTN searches were performed against a reference database, defined by the file of “4090783.454AllContigs.lucy.fa.txt”, which consists of contigs from the ACT-3 metagenome assembled by Newbler v. 2.5. This file can be accessed from IMG taxon object ID of 2100351010 on JGI IMG/m platform (http://img.jgi.doe.gov/cgi-bin/m/main.cgi). To automate BLASTN search, NCBI Blast command line applications should be installed locally and the contig file should be formatted into a nucleotide database using the “makeblastdb” command.
(PL) Click here for additional data file.

## References

[1] Mardis E, McPherson J, Martienssen R, Wilson RK, McCombie WR (2002) What is finished, and why does it matter. Genome Res 12: 669–671. 11997333 10.1101/gr.032102

[2] Gordon D, Desmarais C, Green P (2001) Automated finishing with Autofinish. Genome Res 11: 614–625. 11282977 10.1101/gr.171401 PMC311035

[3] Assefa S, Keane TM, Otto TD, Newbold C, Berriman M (2009) ABACAS: algorithm-based automatic contiguation of assembled sequences. Bioinformatics 25: 1968–1969. 19497936 10.1093/bioinformatics/btp347 PMC2712343

[4] Chain PSG, Grafham DV, Fulton RS, FitzGerald MG, Hostetler J, et al (2009) Genome project standards in a new era of sequencing. Science 326: 236–237. 19815760 10.1126/science.1180614 PMC3854948

[5] Kingsford C, Schatz MC, Pop M (2010) Assembly complexity of prokaryotic genomes using short reads. BMC Bioinformatics 11: 21. 20064276 10.1186/1471-2105-11-21 PMC2821320

[6] Tsai IJ, Otto TD, Berriman M (2010) Improving draft assemblies by iterative mapping and assembly of short reads to eliminate gaps. Genome Biology 11. 10.1186/gb-2010-11-4-r41 PMC2884544 20388197

[7] Boetzer M, Pirovano W (2012) Toward almost closed genomes with GapFiller. Genome Biol 13: R56. 22731987 10.1186/gb-2012-13-6-r56 PMC3446322

[8] Gronow S, Held B, Lucas S, Lapidus A, Del Rio TG, et al (2011) Complete genome sequence of *Bacteroides salanitronis* type strain (BL78). Stand Genomic Sci 4: 191–199. 21677856 10.4056/sigs.1704212 PMC3111984

[9] Peng Y, Leung HCM, Yiu SM, Chin FYL (2011) Meta-IDBA: a *de novo* assembler for metagenomic data. Bioinformatics 27: I94–I101. 21685107 10.1093/bioinformatics/btr216 PMC3117360

[10] Laserson J, Jojic V, Koller D (2011) Genovo: *De novo* assembly for metagenomes. J Comput Biol 18: 429–443. 21385045 10.1089/cmb.2010.0244

[11] Koren S, Treangen TJ, Pop M (2011) Bambus 2: scaffolding metagenomes. Bioinformatics 27: 2964–2971. 21926123 10.1093/bioinformatics/btr520 PMC3198580

[12] Grostern A, Edwards EA (2006) A 1,1,1-trichloroethane-degrading anaerobic mixed microbial culture enhances biotransformation of mixtures of chlorinated ethenes and ethanes. Appl Environ Microbiol 72: 7849–7856. 17056695 10.1128/AEM.01269-06 PMC1694251

[13] Grostern A, Duhamel M, Dworatzek S, Edwards EA (2010) Chloroform respiration to dichloromethane by a *Dehalobacter* population. Environ Microbiol 12: 1053–1060. 20089043 10.1111/j.1462-2920.2009.02150.x

[14] Tang S, Edwards EA (2012) Identification of *Dehalobacter* reductive dehalogenases that catalyze dechlorination of chloroform, 1,1,1-trichloroethane and 1,1-dichloroethane. submitted. 10.1098/rstb.2012.0318 PMC3638459 23479748

[15] Margulies M, Egholm M, Altman WE, Attiya S, Bader JS, et al (2005) Genome sequencing in microfabricated high-density picolitre reactors. Nature 437: 376–380. 16056220 10.1038/nature03959 PMC1464427

[16] Drummond AJ, Ashton B, Buxton S, Cheung M, Cooper A, et al.. (2011) Geneious v5.4.2. Biomatters, Ltd., Auckland, New Zealand.

[17] Huang W, Marth G (2008) EagleView: a genome assembly viewer for next-generation sequencing technologies. Genome Res 18: 1538–1543. 18550804 10.1101/gr.076067.108 PMC2527701

[18] Lapidus A, Labutti K, Foster B, Lowry S, Trong S, et al.. (2008) POLISHER: An effective tool for using ultra short reads in microbial genome assembly and finishing. Advances in Genome Biology and Technology. Marco Island, FL.

[19] Simmons SL, Dibartolo G, Denef VJ, Goltsman DS, Thelen MP, et al (2008) Population genomic analysis of strain variation in *Leptospirillum* group II bacteria involved in acid mine drainage formation. PLoS Biol 6: e177. 18651792 10.1371/journal.pbio.0060177 PMC2475542

[20] García Martín H, Ivanova N, Kunin V, Warnecke F, Barry KW, et al (2006) Metagenomic analysis of two enhanced biological phosphorus removal (EBPR) sludge communities. Nat Biotechnol 24: 1263–1269. 16998472 10.1038/nbt1247

[21] Ettwig KF, Butler MK, Le Paslier D, Pelletier E, Mangenot S, et al (2010) Nitrite-driven anaerobic methane oxidation by oxygenic bacteria. Nature 464: 543–548. 20336137 10.1038/nature08883

[22] Zerbino DR, Birney E (2008) Velvet: algorithms for de novo short read assembly using *de Bruijn* graphs. Genome Res 18: 821–829. 18349386 10.1101/gr.074492.107 PMC2336801

[23] Butler J, MacCallum I, Kleber M, Shlyakhter IA, Belmonte MK, et al (2008) ALLPATHS: *de novo* assembly of whole-genome shotgun microreads. Genome Res 18: 810–820. 18340039 10.1101/gr.7337908 PMC2336810

[24] Simpson JT, Wong K, Jackman SD, Schein JE, Jones SJ, et al (2009) ABySS: a parallel assembler for short read sequence data. Genome Res 19: 1117–1123. 19251739 10.1101/gr.089532.108 PMC2694472

[25] Boetzer M, Henkel CV, Jansen HJ, Butler D, Pirovano W (2011) Scaffolding pre-assembled contigs using SSPACE. Bioinformatics 27: 578–579. 21149342 10.1093/bioinformatics/btq683

[26] Iverson V, Morris RM, Frazar CD, Berthiaume CT, Morales RL, et al (2012) Untangling genomes from metagenomes: revealing an uncultured Class of marine *Euryarchaeota* . Science 335: 587–590. 22301318 10.1126/science.1212665

