# Supplementary material for: Semi-Automatic In Silico Gap Closure Enabled De Novo Assembly of Two Dehalobacter Genomes from Metagenomic Data
Source: PLoS One. 2012 Dec 21;7(12):e52038. doi: 10.1371/journal.pone.0052038 (PMC3528712; doi:10.1371/journal.pone.0052038)
Supplement: Figure S1 — Visualization of raw reads suppressed at the 5′ edge of contig00270. Each row is a raw read. The raw reads that were suppressed (but match each other) are highlighted in red. (DOCX) [file pone.0052038.s001.docx]

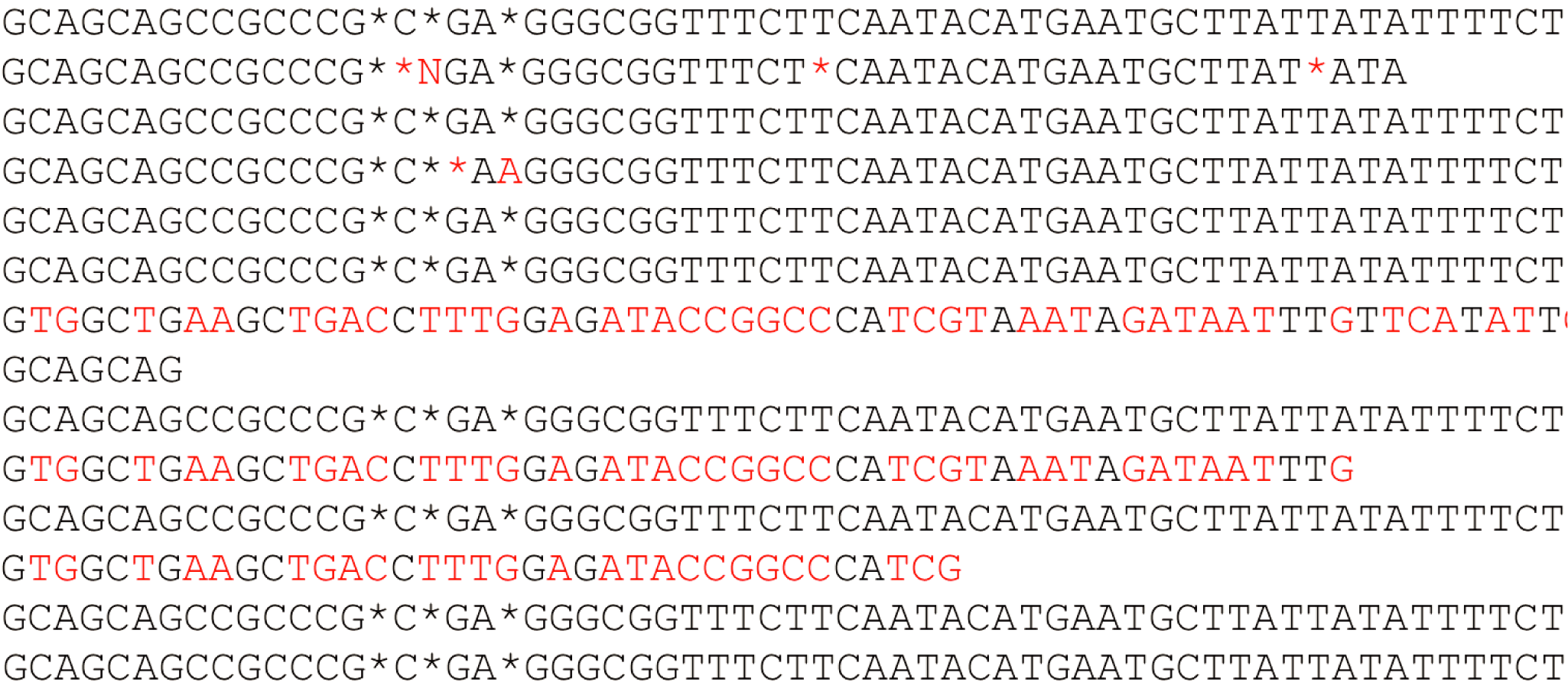


**Figure S1.** **Visualization of raw reads suppressed at the 5’ edge of contig00270.** Each row is a raw read. The raw reads that were suppressed (but match each other) are highlighted in red.
