# Supplementary material for: Semi-Automatic In Silico Gap Closure Enabled De Novo Assembly of Two Dehalobacter Genomes from Metagenomic Data
Source: PLoS One. 2012 Dec 21;7(12):e52038. doi: 10.1371/journal.pone.0052038 (PMC3528712; doi:10.1371/journal.pone.0052038)
Supplement: Figure S2 — Detection of tandem repeats by read mapping. The vertical line in the middle indicates the region of poly-N sequence (50 bp) that is inserted between the tandem copies of the transposase gene related to contig01504. Although the coverage in this region is zero, many read pairs spanning this region were identified, which proves the existence of the tandem copies. (DOCX) [file pone.0052038.s002.docx]

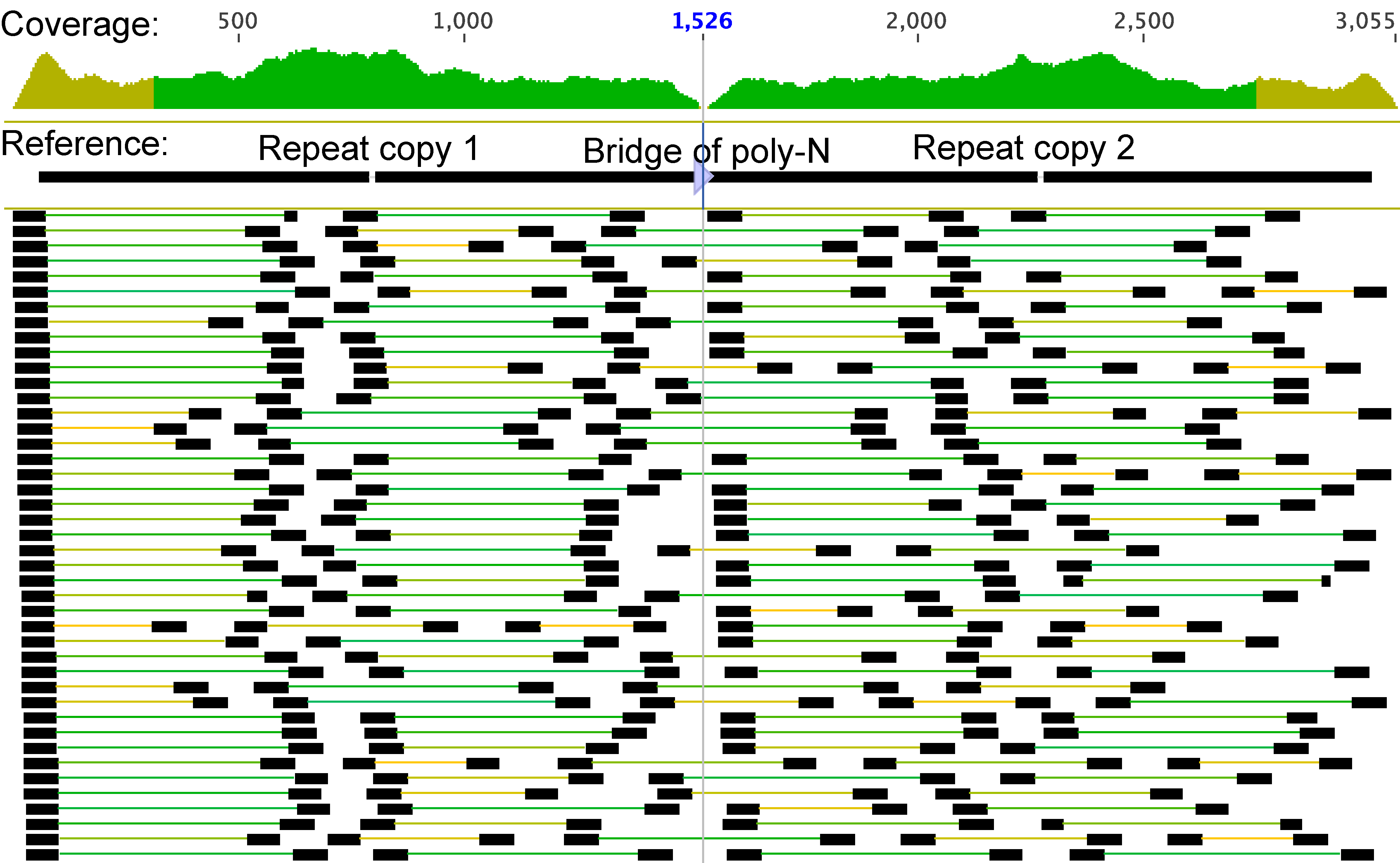


**Figure S2**. **Detection of tandem repeats by read mapping.** The vertical line in the middle indicates the region of poly-N sequence (50 bp) that is inserted between the tandem copies of the transposase gene related to contig01504. Although the coverage in this region is zero, many read pairs spanning this region were identified, which proves the existence of the tandem copies.
