# Supplementary material for: Semi-Automatic In Silico Gap Closure Enabled De Novo Assembly of Two Dehalobacter Genomes from Metagenomic Data
Source: PLoS One. 2012 Dec 21;7(12):e52038. doi: 10.1371/journal.pone.0052038 (PMC3528712; doi:10.1371/journal.pone.0052038)
Supplement: Table S1 — Contigs that contain reads whose sequences were suppressed. (DOCX) [file pone.0052038.s003.docx]

**Table S1. Contigs that contain reads whose sequences were suppressed**

|  | Suppressed Sequences | | | | |
| --- | --- | --- | --- | --- | --- |
| Contig ID | 5’ Edge | | 3’ Edge | | |
|  | Representative Read Name | No.^1^ | Representative Read Name | No. ^1^ |  |
| 00230 |  |  | GJDNVXK02FK38E | 8 |  |
| 00241 |  |  | GJDNVXK02I4DG4 | 6 |  |
| 00242 | GJDNVXK02GH51R | 13 |  |  |  |
| 00243 |  |  | GJDNVXK02GYCR5 | 7 |  |
| 00244 |  |  | GJDNVXK01BIEUN | 13 |  |
| 00245 | GQIUW4001DHH63_left | 10 |  |  |  |
| 00246 | GQIUW4001D6TGY_right | 10 |  |  |  |
| 00247 |  |  | GJDNVXK01COW8I | 12 |  |
| 00248 |  |  | GJDNVXK01DDO4A | 12 |  |
| 00249 | GJDNVXK02I9OPG | 6 |  |  |  |
| 00252 | GQIUW4002HJYIK | 11 |  |  |  |
| 00253 |  |  | GQIUW4002IKBAU | 12 |  |
| 00255 |  |  | GJDNVXK01A2VBS | 12 |  |
| 00259 | GJDNVXK01EQO8R | 11 | GJDNVXK02H78LB | 6 |  |
| 00260 | GJDNVXK01C8EDU | 11 |  |  |  |
| 00265 | GJDNVXK01BT6ZY | 6 |  |  |  |
| 00269 | GJDNVXK01EVGRK | 8 |  |  |  |
| 00270 |  |  | GJDNVXK01E3JVP | 12 |  |
| 00272 | GQIUW4002GKUZE | 7 |  |  |  |
| 00274 | GJDNVXK02G4J27 | 22 |  |  |  |
| 00279 | GJDNVXK01AC3MK | 8 |  |  |  |
| 00284 | GJDNVXK02FVHEJ | 2 |  |  |  |
| 00288 |  |  | GJDNVXK01CTGJ1 | 16 |  |
| 00289 | GJDNVXK02I3ZUD | 3 | GJDNVXK02IAXE9 | 9 |  |
| 00290 |  |  | GJDNVXK02F6K62 | 12 |  |
| 00299 |  |  | GQIUW4001BSRMU | 13 |  |
| 00302 |  |  | GJDNVXK01EO1PM | 24 |  |
| 00309 | GQIUW4002IEBPH | 12 |  |  |  |
| 00530 | GJDNVXK02FN2A2 | 16 |  |  |  |
| 00539 |  |  | GQIUW4002GSV6F_right | 9 |  |
| 00540 |  |  | GJDNVXK02GPLN6 | 14 |  |
| 01315R | GJDNVXK02FIYJF | 23 | GJDNVXK02J2VE2 | 25 |  |
|  |  |  | GJDNVXK01ANVGN | 34 |  |
| 05122 | GJDNVXK01DTW10 | 19 |  |  |  |
|  | GJDNVXK02IJB8Z | 23 |  |  |  |
| 01997 |  |  | GJDNVXK01AYWWU | 23 |  |

^1^ The number of raw reads that were suppressed but had sequences homologous to the representative suppressed read listed.
