# Supplementary material for: Semi-Automatic In Silico Gap Closure Enabled De Novo Assembly of Two Dehalobacter Genomes from Metagenomic Data
Source: PLoS One. 2012 Dec 21;7(12):e52038. doi: 10.1371/journal.pone.0052038 (PMC3528712; doi:10.1371/journal.pone.0052038)
Supplement: Table S2 — Experimental verification of the resolution of 22 assembly gaps. (DOCX) [file pone.0052038.s004.docx]

**Table S2. Experimental verification of the resolution of 22 assembly gaps**

| Repetitive Contigs | Gaps | PCR Amplicon Size | | Sanger Sequencing^3^ | Primers | |
| --- | --- | --- | --- | --- | --- | --- |
|  |  | Predicted^1^ | Experimental^2^ |  |  |  |
| 01388 | 00253-G-00254 | 2470 | 2400 | + | F | TCCGCTCCATAGGCACCTCGT |
|  |  |  |  |  | R | CCGTCCGACAAGCTCAAAACGG |
|  | 00268-G-00269 | 2282 | 2200 | + | F | AGTGGCACGTAGCGTTGAACA |
|  |  |  |  |  | R | TCGACGATTTCCGCTTGTTGCT |
|  | 00278-G-00279 | 2429 | 2400 | + | F | AGCCTGCCCTTTGGAGAAAGACA |
|  |  |  |  |  | R | CGGCAACAGGCTTGTCGGCAT |
|  | 00538-G-00539 | 2251 | 2200 | + | F | TGGGCTATTATAGTCAGCGGCGT |
|  |  |  |  |  | R | TTGGACTCGTGGGGTGGAACT |
|  | 00232-G-00233 | 3252 | 3100 | + | F | TCGTCTCCACTCATTATCCCGGC |
|  |  |  |  |  | R | GCGGAAAAATTGTCCTGGCCACA |
|  | 00251-G-00252 | 2415 | 2500 | - | F | AGGAGATTGCTGATGCGGTGGGA |
|  |  |  |  |  | R | AAGCCGTTAGGTGTGCCCGC |
|  | 00310-G-00311 | 2970 | 2900 | + | F | TCCAGTTCAACATCGGCTGTGCT |
|  |  |  |  |  | R | GTTTGAGTGTCCTCCTGGGCTGA |
| 01504 | 00265-G-00266 | 1746 | 1750 | + | F | ATGTTCATTTTCGGGGCCGTTGA |
|  |  |  |  |  | R | GCGAGCGCCTTCGACCAACT |
|  | 00266-G-00267 | 2005 | 2000 | + | F | TCATGCCCCTGAAGTCGCGG |
|  |  |  |  |  | R | TGCACTCCTCCTGTCTGGTACG |
|  | 00267-G-00268 | 1852 | 1750 | + | F | TGGCATTTTGCGCTGGCTGG |
|  |  |  |  |  | R | TGGTCTTGGCCCTTGCGAGC |
|  | 00285-G-00286^4^ | 1910/3384 | 1900/3400 | + | F | GCAATGTTTTGCGCCTTGGTGA |
|  |  |  |  |  | R | TGGCAGGGGATGAAGGAGTTGA |
|  | 00313-G-00314 | 2264 | 2200 | + | F | CGGTGGTTTGGCAGCAGGA |
|  |  |  |  |  | R | GGGTCCAAGAAATGGCGGAAG |
|  | 00242-G-00243 | 1774 | 1800 | + | F | ACCCCAACAGCTTTCAGACGGGT |
|  |  |  |  |  | R | CGGACGCAATCTCTCAGCATTCG |
|  | 00314-G-00228 | 1803 | 1800 | + | F | TCCCGCCCCGAGACGCTTTA |
|  |  |  |  |  | R | CGGGTCAATCATCGGCGGAGT |
|  | 00268-G-00269 | 2017 | 2000 | + | F | TCCTGCCTTCGATAAAAGCCTGT |
|  |  |  |  |  | R | AGGCGAAAGAACCGGCGTACA |
| 01532 | 00269-G-00270 | 2461 | 2300 | + | F | AAGTTGCCGCTGCTGTCGCT |
|  |  |  |  |  | R | TGTCCAACCTAAATGCCGCCGA |
|  | 00274-G-00275 | 2238 | 2100 | + | F | GGAACCCATCCTGTGACCGT |
|  |  |  |  |  | R | GCGCGAACGATATGCCAAAGGG |
|  | 00279-G-00280 | 2391 | 2500 | + | F | GCGCTGACGTTGTGCCTGAA |
|  |  |  |  |  | R | AGTAGTGCCGGGGGTTAGTGT |
|  | 00239-G-00240^4^ | 2579/4476 | 2500/4200 | + | F | ACGGCATTTGAACCTGAAGGCCA |
|  |  |  |  |  | R | GCCAAGGGAATCCGGCGGTC |
|  | | | | | | |
| Group A Gaps | 00277-G-00278 | 968 | 1000 | + | F | GAAAGGAGGCCGCAGTCCG |
|  |  |  |  |  | R | GCCTGACGAACCGTGGATTGAT |
|  | 00284-G-00285 | 1006 | 1000 | + | F | TCCGCTCCCCGTATGCCCT |
|  |  |  |  |  | R | TGCCCGAAAAGCGAAAAGCGT |
|  | 00298-G-00299 | 1065 | 1100 | + | F | GCCCATAGGTGGCGTCGATGA |
|  |  |  |  |  | R | TGGCAGAGGGGAAGCTCAGGG |

^1^ The predicted amplicon size was calculated based on the solution from current gap-resolution strategy.

^2^ The experimental amplicon size was determined by performing DNA electrophoresis and comparing the DNA bands with DNA ladders.

^3^ Partial sequencing of the amplicons using Sanger sequencing: “+” indicates that the DNA sequence determined by Sanger sequencing matches the expected sequence of the amplicon determined by *in silico* sequence assembly; “-” indicates that Sanger sequencing failed with unknown reason.

^4^ For gap 00285-G-00286 and gap 00239-G-00240, in which tandem repeats were expected, two PCR products with different size were found amplified simultaneously.
